# The Treatment of Acute Coryza

**Published:** 1912-11-30

**Authors:** 


### THE TREATMENT OF ACUTE CORYZA.

The common cold in the head is one of the minor ills of life to which most people are liable. Garel advises that at the commencement such general treatment as is productive of sweating should be employed, as, for example, mustard foot-baths, tincture of aconite, Dover's powder, or Mindererus' spirit; and to arrest its progress he advises inhalations of hot water for five minutes at a time, three or four times a day, after painting the nasal mucous membrane with two or three drops of 1 in 1,000 solution of adrenalin. In the adult, $\frac{1}{200}$ grain of atropine sulphate, morning and evening, will check the excessive nasal secretion. As abortive treatment a nasal douche of lemon juice, repeated twice, usually suffices. Roux prefers eau-de-Cologne for the purpose. Rubbing of the nose between the thumb and forefinger, by increasing the hyperæmia of the mucous membrane, sometimes acts wonderfully. Hayem advises that a few drops of the following mixture be poured on blotting-paper and the vapour inhaled for a few seconds:

| | |
|---|---|
| Carbolic acid, pure ... ... ... | 1 part |
| Liquid ammonia ... ... ... | 1 part |
| Alcohol ... ... ... ... ... | 2 parts |
| Distilled water... ... ... ... | 3 parts |

Such inhalations should not be abused, because they may be productive of aural complications. Equal parts of camphor and bismuth subnitrate are recommended as a snuff. The nasal obstruction may be more advantageously dealt with by the use of cocaine, in such a prescription as follows:

| | |
|---|---|
| Cocaine hydrochlor. ... ... ... | gr. j. |
| Menthol ... ... ... ... ... | gr. j. |
| Roasted and powdered coffee ... | ʒ j. |
| Acid, boric ... ... ... ... | ʒ v. |

Here the action of the cocaine, which is rapid but of short duration, is prolonged by the addition of menthol, and to render the powder more palpable so as to permit of its being easily snuffed the powdered coffee is added. A similar snuff, in which salol takes the place of the cocaine, dispenses with the powdered coffee.

In cases where, on account of the nasal obstruction, it is difficult for the patient to insufflate the powder, Garel recommends that a piece of india-rubber tubing about eight inches long should be used: the powder is placed in one end of the tube, which is held at the nasal orifice, whilst the patient blows at the other end and distributes it over the mucous membrane.

But, perhaps, the treatment that gives the most relief is adrenalin. A brush should be dipped in two or three drops of 1 in 2,000 adrenalin solution and the Schneiderian membrane lightly touched with it. With this cocaine may be combined. An agreeable inhalation may be made from decoction of verbena, to which is added a teaspoonful of a solution of menthol in olive oil. The febrile symptoms may temporarily be checked by means of a spray of antipyrin 2 to 4 in 1,000, whilst the previous application of cocaine prevents the tingling and burning of the mucous membrane set up by the antipyrin.

When inflammation arises on the lips or at the nasal orifice the following ointment will be found useful:

| | |
|---|---|
| Cocaine hydrochlor. ... ... ... | gr. j. |
| Tannin... ... ... ... ... | gr. 10 |
| Cold cream ... ... ... ... | gr. 40 |

